# Tuberculosis infection related hemophagocytic lymphohistiocytosis diagnosed in patient with GZMB mutation: A case report and literature review

**DOI:** 10.1097/MD.0000000000030283

**Published:** 2022-09-02

**Authors:** Zhenhao Wang, Jin Zhang, Zhaonian Hao, Li Meng, Zhiqiang Han, Zhenya Hong

**Affiliations:** a Department of Hematology, Tongji Hospital, Tongji Medical College, Huazhong University of Science and Technology, Wuhan, 430030, P.R.China; b The Second Clinical School Affiliated Tongji Hospital, Tongji Medical College, Huazhong University of Science and Technology, Wuhan, 430030, P.R.China; c Cancer Biology Research Center (Key Laboratory of the Ministry of Education), Tongji Hospital, Tongji Medical College, Huazhong University of Science and Technology, Wuhan, 430030, P.R.China

**Keywords:** GZMB, Hemophagocytic lymphohistiocytosis, Metagenomic next-generation sequencing, *Mycobacterium tuberculosis*

## Abstract

Secondary hemophagocytic lymphohistiocytosis (HLH) is a life-threatening syndrome associated with infections, tumors and connective tissue disease. However rapid identification of the underlying infectious cause of HLH is challenging because traditional etiological diagnostics are time-consuming and sometimes fail to identify the pathogens. Metagenomic next-generation sequencing (mNGS) may be a potential optimal solution, which may help improve the clinical diagnosis of underlying infections in hematological diseases.

**Case presentation:**

A 28-year-old man presented with a 2-month history of intermittent fever and cytopenia. The HLH was diagnosed based on the manifestations of fever, splenomegaly, anemia, thrombocytopenia, hyperferritinemia, hyperglyceridemia, and elevated IL-2R levels. High-through-put sequencing analysis detected a GZMB mutation. While the initial detection of cultures and smears of tuberculosis was negative, TB infection was eventually identified by mNGS of blood sample. The symptoms rapidly abated during the initial administration of TB.

**Conclusion:**

The present case proposed that mNGS might be an effective diagnostic tool for diagnosing rare infectious cause of secondary HLH. GZMB mutation was first discovered to be present in secondary HLH.

## 1. Introduction

Hemophagocytic lymphohistiocytosis (HLH) is a potential life-threatening monocyte-macrophage related histiocytic disorder, which results in a series of clinical signs and symptoms characterized primarily by fever, hepatomegaly, splenomegaly and pancytopenia.^[1]^ HLH can be categorized into primary and secondary types according to different triggering factors. Primary or familial HLH commonly presents in young children with specific gene mutations or a family history of HLH. Secondary or acquired HLH can be caused by a variety of pathogenic processes, including infection, malignancy, autoimmune disease and drug hypersensitivity reaction, which may occur at any age. A wide variety of infections may be associated with HLH, including TB.^[2–4]^ HLH is an aggressive and fatal syndrome, early identification of cause and management of HLH is crucial, particularly in HLH associated with nonviral infections, as underlying infectious agents often have good respond to antimicrobial treatment.^[5]^

The emerging metagenomic next-generation sequencing (mNGS) technology, can be possible solution to improve the diagnostics. mNGS is based on the meta genome and not depending on the traditional microbial cultures. The pathogenic microbe can be identified according to the result of comparison on the sequence. Therefore, mNGS is considered to have the ability of producing rapid, objective and broad-range detection of pathogenic microbes (including viruses, bacteria, fungi, parasites) in a single assay, which makes it suitable for the diagnosis of acute, severe and intractable infection.^[6,7]^

Here we present a case of TB related HLH diagnosed by mNGS in adult patient with GZMB mutation. While conventional diagnostics failed to confirm the suspected TB infection, mNGS subsequently confirmed the underlying TB infection, which led to initiation of anti-TB therapy and the patient gained rapid clinical response and disease remission.

## 2. Case presentation

A 28-year-old male laborer was admitted to the department of infectious disease of our hospital with a 2-month history of intermittent fever with shivers, anemia and thrombocytopenia. During the previous month, the patient complained of fatigue and decreasing of appetite, as well as a 2kg weight loss. antiinfection treatment was given in local hospital, along with a suspected diagnosis of TB infection and atypical chronic myeloid leukemia, but had poor response. No remarkable medical history was reported, and the patient declared no cigarette and illicit drugs usage. There was no family history of immunodeficiencies, autoimmune diseases nor early childhood deaths. Notably, the patient was employed as a laborer and lived in a dormitory with poor living condition.

On physical examination, His baseline vital signs included a temperature of 38.3°C, a blood pressure of 95/59 mm Hg and a heart rate of 137bpm (tachycardia on ultrasonic cardiogram). Cardiovascular and pulmonary examinations were unremarkable. Abdominal examination revealed splenomegaly, and neck examination found a swelled lymph node at left neck.

**Figure 1.**
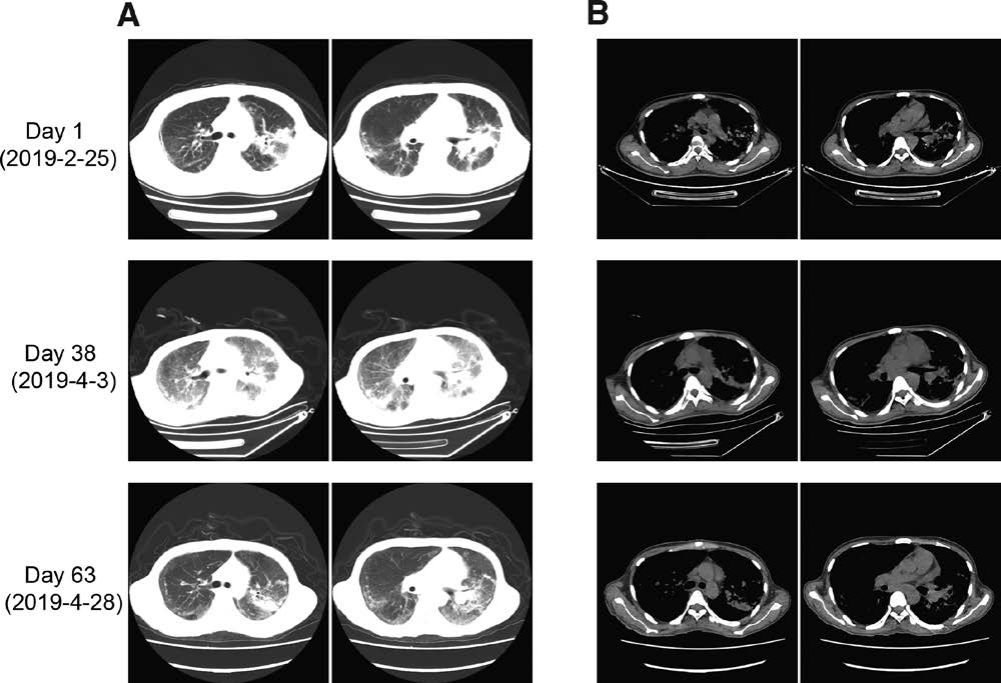
Chest CT of patient at admission, day 38 and day 63. (A) Lung window. (B) Mediastinal window.

**Figure 2.**
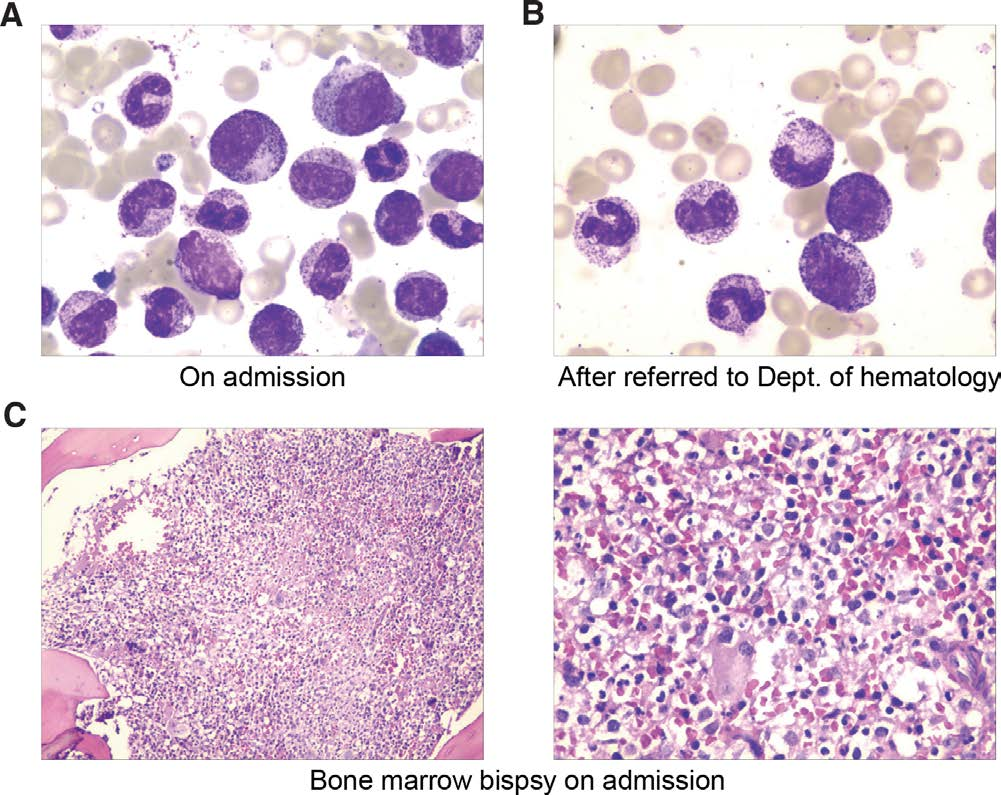
Cytological analysis of bone marrow samples. (A) Hematoxylin and eosin (H&E) staining of bone marrow aspirate slides on admission. (B) H&E staining of bone marrow aspirate slides after referred to department of hematology. (C) Histological analysis of bone marrow sections by H&E staining on admission.

Bone marrow smear and biopsy in local hospital suggested a suspected diagnosis of chronic myeloid leukemia with a proliferated myeloid series. Baseline laboratory evaluation revealed normocytic anemia, a hemoglobin level of 72 g/L (130–175g/L), a platelet count of 94 × 10^9^/L (125-350 × 10^9^/L) and a raised white cell count of 13.77 × 10^9^/L (3.5-9.5 × 10^9^/L), including 93.8% neutrophils. Liver function tests were deranged with mildly raised alanine aminotransferase 57 U/L (≤41 U/L), raised aspartate aminotransferase 73 U/L (≤40 U/L), raised alkaline phosphatase 293U/L (40–130 U/L), raised γ-glutamyl transferase 511U/L (10–71U/L), raised lactate dehydrogenase 768 U/L (135–225 U/L), raised triglycerides 2.4 mmol/L (normal < 1.7 mmol/L), and mildly decreased albumin 31.5 g/L (35–52 g/L). Renal function tests were within normal threshold. Tests for acute phase reactants revealed raised C reactive protein 83.5 mg/L and raised serum ferritin 7688.7 ug/L (30–400 ug/L), and his serum IL-2R level was 2260U/ml (223–710U/ml). Clotting test revealed raised fibrinogen 5.88g/L (2.00–4.00g/L) and prolonged activated partial thromboplastin time (APTT) 44.9 s (29–42s). And baseline screening for pathogens was negative (bacteriological cultures of blood, sputum and bone marrow were negative, while multiple induced sputum smear samples were negative for acid-fast organisms, and the serum G/GM test were negative.). CT scan of his chest reveals both lungs infiltrates with scattered multiple stripes, patch shadows and nodules, bilateral pleural thickening and adhesions, small amount of bilateral pleural effusion and pericardial effusion, increased mediastinal lymph nodes and splenomegaly (Fig. 1A). Ultrasonic examination of abdomen revealed hepatomegaly and splenomegaly. The bone marrow smear showed a proliferated myeloid series with left shift and toxic changes, while no abnormal cell was found; bone marrow biopsy showed proliferated myeloid series and megakaryocyte could be seen 2~9/HPF (Fig. 2A,C). Both the bone marrow aspirate and biopsy did not find hemophagocytosis. No immunophenotypic evidence of a lymphoproliferative disease by flow cytometric analysis and no monoclonal component was identified. In addition, serum investigations were negative for HIV, hepatis C antibody and treponema pallidum antibody. Hepatitis B surface antibody, hepatitis B e antibody, and hepatitis core antibody were positive. T-spot test showed negative result of TB specific immune response. DNA copies of cytomegalovirus were below detection limit, while DNA copies of Epstein-Barr virus were lower than 500 copies/ml. Considering his polyserous effusions, tests for connective tissue diseases were administrated. The results showed that antinuclear antibodies, liver-kidney microsomal antibody and antineutrophil cytoplasmic antibodies were all negative, excepting the antismooth muscle antibody. Hemolytic and nutritional anemia tests were all within normal range.

The patient’s hospital course was notable for intermittent fevers as high as 40°C and worsening anemia. During the first 25 days in his hospitalization, the patient received antiinfection treatment including moxifloxacin, amikacin, tigecycline and linezolid, but did not alleviate his symptoms. The reexamination of chest CT scan on day 15 showed enlarged pulmonary nodules and worse pericardial effusion. The patient experienced a nadir hemoglobin level of 37 g/L, and underwent several transfusions of red cells. According to the manifestations of fever, splenomegaly, anemia and thrombocytopenia, hyperferritinaemia, hyperglyceridemia and elevated IL-2R, the diagnosis of hemophagocytic syndrome was made on the basis of the HLH-2004 diagnosis criteria.^[1]^ The patient was then referred to the department of hematology.

Another bone marrow aspirate was performed in the hematology ward. The karyotype analysis revealed a normal 46, XY male karyotype, and there was still no immunophenotypic evidence was found by flow cytometric analysis. BCR/ABL fusion gene and MPN related genes had not been detected. No significant change could be found from bone marrow smear with the previous one (Fig. 2B). Then a fine-needle aspirate (FNA) was performed on the enlarged cervical lymph node, but histopathology of the lymph node didn’t support the diagnosis of lymphoma or TB. In the purpose of a clear-cut diagnosis, a high-throughput sequencing (using Ion Torrent PGM/Illumina NextSeq 550Dx Platform) of HLH related gene was applied (Table 1), and a missense mutation in exon 3 of GZMB gene was identified, c.314T > G, p.Phe105Cys (p.F105C). We assume that the mutation of GZMB in the patient may results in the decline of the cytotoxicity of his lymphocytes and be associated with the hemophagocytic lymphohistiocytosis.

**Table 1:** Screen for HLH associated genes by high-throughput sequencing.

Screened genes					
LYST, CTPS1, PIK3CD, PRF1, SRGN, CD27, LAMP1, ARF6, GZMB, RAB27A, BLOC1S6, CORO1A, UNC13D, STXBP2, GNLY, STK4, PRKCD, AP3B1, ITK, STX11, CARD11, MCM4, MAGT1, SH2D1A, XIAP, IL2RD					
Sequencing finding					
Locus	Gene	Mutation	Type	Ratio (%)	Coverage
14:25101555	GZMB	c.314T > G	Missense	49.5	489
NM_004131.5		p.Phe105Cys			

**Figure 3.**
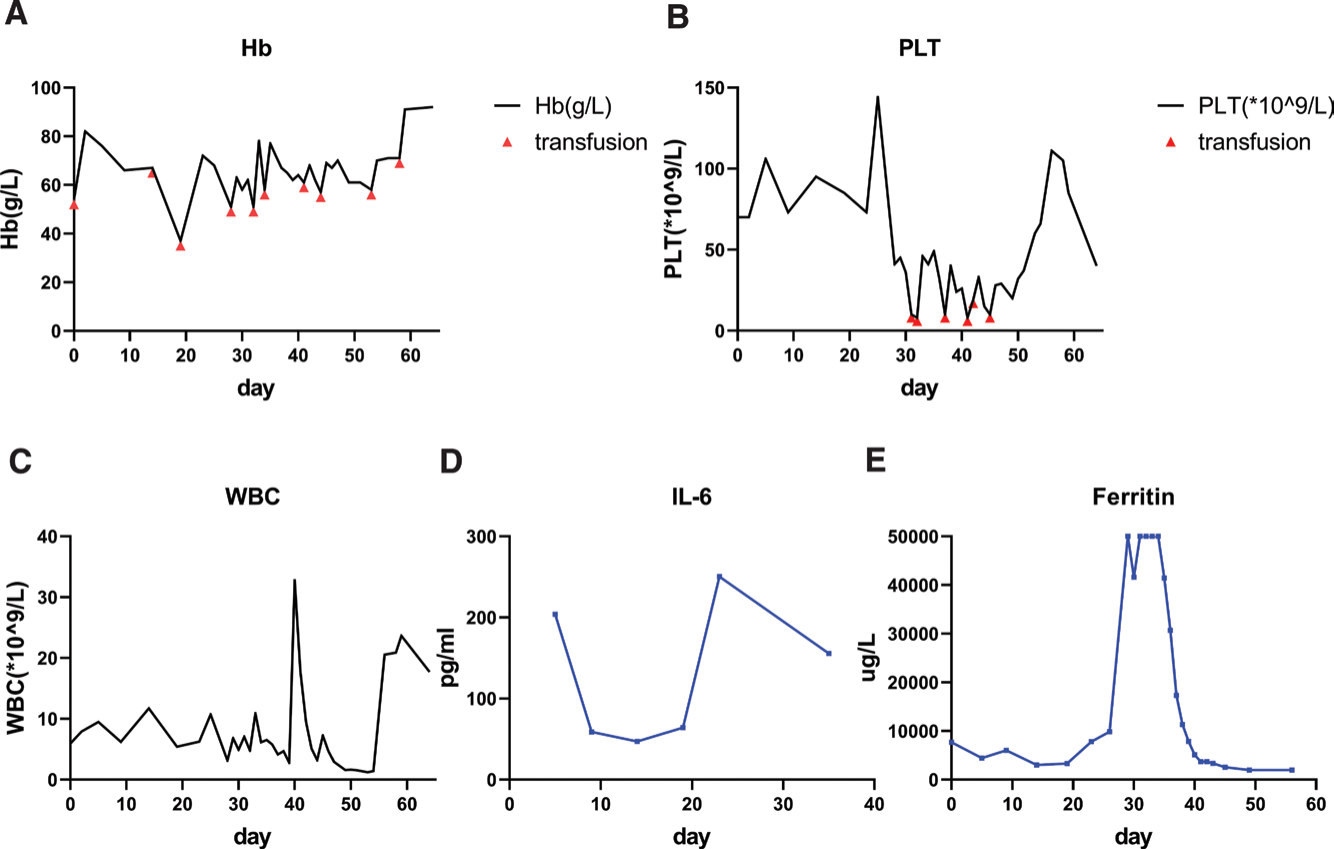
Hemogram and laboratory tests monitoring showed the patient largely improved. (A–C) Hb, platelet and WBC level changes during treatment. (D) and (E) Level of IL-6 and ferritin changes during treatment.

Empirical antibiotic therapy started at day 26 since hospitalization, including teicoplanin, meropenem, moxifloxacin and voriconazole. The patient’s condition deteriorated by the day 28 as brisk drop of platelet, steep elevation of ferritin and possible development of disseminated intravascular coagulation (ferritin above 50000 ug/L, D-dimer 42.39 ug/ml, APTT 48.1s, 41 × 10^9^ platelet per L, fibrinogen 1.72 g/L, procalcitonin 2.09 ng/ml), the patient got transfusion dependency (red cells and platelets) (Fig. 3A,B). Another chest CT scan on day 29 showed some exacerbation of his pulmonary infection and worse pleural and pericardial effusion. Treatment was initiated with dexamethasone 10 mg and gamma immunoglobulin 5 g intravenously on day 29, and following by a 4-day therapy of higher dose of immunoglobulin (20 g intravenously daily). Meanwhile teicoplanin, meropenem, moxifloxacin and voriconazole were discontinued, multiple courses of antimicrobials (including tigecycline, linezolid, cefoperazone/sulbactam, caspofungin and ganciclovir) were administrated to control his infection. A total of 1250ml fresh frozen plasma, 400ml cryoprecipitate and 1g fibrinogen were infused during day 29 to 34 to correct his coagulation abnormalities. Given the history of poor living condition in dormitory and emaciation, empirical oral isoniazid 0.2 g daily was given started from day 37 despite of no definitive evidence of TB. Etoposide was given on hospital day 33–35 and day 39 (0.1 g intravenously daily), and dexamethasone was administrated 15 mg intravenously daily for a week (day 31–38), then reduced to10 mg daily for 3 days, and reduced to 7.5 mg daily for another week (day 42–49), 5 mg daily for day 50–57, and which finally tapered to 30 mg oral prednisone daily. On day 38, a chest CT scan showed aggravated infection than the previous CT (Fig. 1), but his serum ferritin and procalcitonin level decreased to 11308.0 ug/L and 0.61 ng/ml, respectively (Fig. 3E).

**Figure 4.**
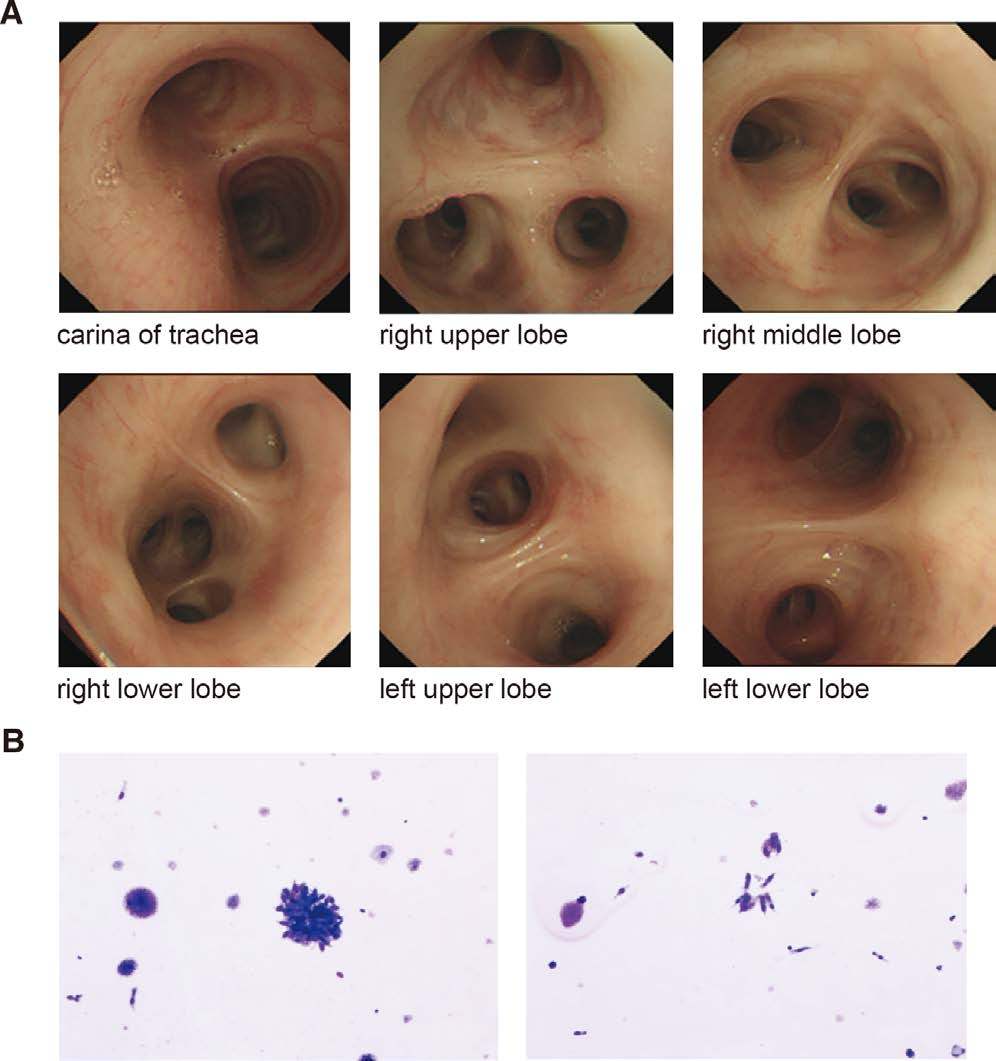
Bronchoscope and liquid-based cytology for bronchial lavage. (A) Bronchoscope found bilateral bronchial inflammatory changes. (B) Liquid-based cytology showed only ciliated columnar epithelium cells and macrophages.

On day 39, his blood sample was sent to Wuhan BGI Co. Ltd. for mNGS. mNGS detected sequences of TB complex in the sample (Table 2) within 48 hours of the sample receipt. After 7-day application of isoniazid, the patient’s condition improved, as he had no fever and abated symptoms, the laboratory tests showed a WBC count of 4.73 × 10^9^/L with 95.6% neutrophils and 4% lymphocytes, a hemoglobin level of 67.0 g/L, platelet count of 28 × 10^9^/L, and serum ferritin level decreased to 2552.6 ug/L. Tigecycline and ganciclovir were discontinued, and caspofungin was replaced by voriconazole (0.2 g bid intravenously). To confirm the NGS finding of TB existence, on day 60, a fiberoptic bronchoscopy and bronchial lavage (BL) was performed. No other special finding on fiberoptic bronchoscopy than bilateral bronchial mucosal edema. Plenty of translucent secretions on the left bronchus were found, which were suctioned clear and sent for cultures for bacteria, fungus and tuberculosis (Fig.4). BL fluid was sent for examinations including organism cultures, acid-fast stain, GeneXpert, liquid-based cytology and flow cytometry. The cultures turned out negative, and the acid-fast stain found nothing, liquid-based cytology only revealed a few ciliated columnar epithelium cells and macrophages, flow cytometric analysis found no evidence of immunophenotypic abnormity. However, nucleotide of TB and rifampicin resistance gene were detected from GeneXpert. Combining this finding with the NGS report, the diagnosis of TB infection was confirmed. On day 61, an electronic colonoscope was administrated to rule out possible intestinal tuberculosis infection that may lead to the HLH, which turned out no organic lesions in his intestines. On day 63, the CT scan showed the lesions in his lungs were gradually absorbed, and there were no pleural effusions anymore (Fig.1). Before discharge, the patient’s anemia and thrombocytopenia were relieved, fever was also vanished. Blood routine test showed a hemoglobin level of 92 g/L and a platelet count of 40 × 10^9^/L, biochemical and clotting tests showed a normal liver function, and his INR was 0.92, APTT and fibrinogen levels were within normal range. His serum ferritin level had decreased to 1989.4ug/L, which was 74% lower than admission. Based on the HLH-2004 standard, he was evaluated as clinical response. On discharge, he was administrated with anti-TB medication including isoniazid, ethambutol and moxifloxacin. The patient was then transferred to a specialized tuberculosis hospital, and had largely recovered after underwent standard antituberculosis treatment there.

**Table 2:** NGS report of blood sample.

Genus	
Name	Sequence number^*^
Mycobacterium tuberculosis complex	52

* The sequence number of the strict comparison of the microorganism detected at the level of genus/species.

## 3. Discussion and Conclusion

Here, we present a case of HLH associated with TB infection, that the underlying TB infection had been undetected until a mNGS was performed. The patient’s symptoms were partially controlled by HLH treatment, but his pulmonary infection was exacerbated. Eventually, TB was considered the pathogenic bacteria due to the double detection of TB from mNGS and BL tests. Once given anti-TB treatment, the condition of patient was soon improved.

But whether the onset of HLH was initialed from TB or GZMB mutation alone, or the combination of TB infection and GZMB mutation cannot be definitively determined. However, his continued response and lack of relapse of HLH after complete TB treatment prompted us that TB may have been the primary disease. Granzyme B is encoded by GZMB gene, which is a critical serine protease and one of the most powerful pro-apoptotic granzyme in target cell death induced by cytotoxic T lymphocytes and NK cells.^[8]^ However, previous study does not support granzyme B as candidate genes for familial hemophagocytic lymphohistiocytosis, the pathogenicity of the mutation requires further investigation.^[9]^ To our knowledge, this is the first reported case that mNGS was applied to identify underlying TB infection in HLH associated with TB infection in immunocompetent adult, in addition, it is the first time GZMB mutation was described in secondary HLH.

mNGS, a new DNA/RNA detection method and platform, has been increasingly widely applied in clinical practices of acute severe infections and rare pathogen infections in recent years. Owing to its unbiased, rapid and broad-range detection capability, mNGS has been applied in many complicated infection cases and successfully identified the pathogens, including atypical infection of Legionella spp. and Aspergillus fumigatus, and rare pathogens such as St. Louis encephalitis virus, Cache Valley virus, West Nile virus and Orientia tsutsugamushi.^[10–15]^ In addition to its culture-independent and unbiased nature, mNGS has been shown a higher sensitivity and negative predictive value for pathogen identification and less affected by antibiotic exposure.^[16,17]^ In our case, we used mNGS of a blood sample from the patient detected TB, allowing us to make a definitive diagnosis.

In our review of literature, by the end of April 2022, 9 cases of infection related HLH applying mNGS to identify underlying pathogenic microorganisms have been published (Table 3),^[18–26]^ our case is listed in the table as well. The age of patients range from 4-month-old to 49 years old, and their median age is 31 years old. Most listed patients failed to acquire positive results (50%) or cover the rare pathogens (40%) using the traditional etiological diagnostics (including culture, smear, immunological tests), and in the only positive case,^[25]^ physicians mistook Pneumocystis jirovecii as the cause of symptoms, which was later corrected by mNGS. In these cases, a variety of samples have been used for mNGS, including peripheral blood, serum, plasma, sputum, alveolar lavage fluid and lymph node, which suggests the application of mNGS is accessible and reliable for different clinical scenarios and multiple type of specimens.

**Table 3:** Summary of previous case reports.

Case	Time (year)	Age/Sex	Sample for mNGS	Underlying pathogen	Previous pathogenic tests	Reference
1	2020	9.5 month/F	peripheral blood, sputum	Leishmania	Negative (rk39)	Guo F, et al^[18]^
2	2020	42 y/M	serum	Candidatus Mycoplasma haemohominis	Not covered	Hattori N, et al^[19]^
3	2020	4 months/M	peripheral blood	Mycobacterium tuberculosis	Negative (culture, antibody)	Shi B, Chen M, et al^[20]^
4	2020	48y/F	lymph node	Bartonella henselae	Not covered	Yang T, Mei Q, et al^[21]^
5	2021	49y/M	plasma	Human Herpesvirus 8	Not covered	Cui X, et al^[22]^
6	2021	25y/M	peripheral blood	Leishmania	Negative (bone marrow smear)	Lin Z, et al^[23]^
7	2021	27y/F Pregnancy	peripheral blood, alveolar lavage fluid	Chlamydia psittaci	Not covered	Sun L, Li P, et al^[24]^
8	2022	33y/M	plasma	Toxoplasma gondii	Positive (antibody)	Zhou Y, et al^[25]^
9	2022	35y/M	peripheral blood	Severe fever with thrombocytopenia syndrome virus	Not covered	Zhu T, et al^[26]^
This case	2019	29y/M	peripheral blood	Mycobacterium tuberculosis	Negative (culture, antibody)	

This case highlights the importance of applying mNGS as a rapid, sensitive and promising assay for underlying infectious pathogens in the management of patients with infection related HLH. Early initiated treatment based on early diagnosis can reduce mortality both form HLH as well as its underlying infection. Deploying mNGS may help clinicians to make prompt and precise diagnosis of infectious diseases, particularly in immunocompromised hematological patients in the near future. In addition, GZMB mutation in secondary HLH was described in our case.

## Author contributions

Z.W. collected and analyzed the date; Z.W., J.Z., ZN.H., ZY.H., L.M. took care of the patient, provided clinical information. Z.W. wrote the manuscript. ZY.H. and ZQ.H. directed the report. All authors have read and approved the manuscript.

## Acknowledgment

The authors would like to thank all members of the study team, the patient, and his family.
